# Defining difficult public health facilities

**DOI:** 10.1186/1753-6561-6-S5-O14

**Published:** 2012-09-28

**Authors:** Jhimly Baruah, BM Prasad, Anuradha Jain

**Affiliations:** 1 National Health System Resource Centre, Delhi, India

## Introduction

In its efforts to strengthen the rural public health system, the National Rural Health Mission had to address the challenge of getting skilled health care providers to work in rural and remote areas. Learning from states, which had some measure of success with incentives for doctors in such areas, the central government decided to launch a scheme to provide these incentives to states. States were asked to prepare a list of difficult, most difficult and inaccessible facilities based on stated criteria for defining ‘difficulty’. However, these criteria evolved by states were often subjective and could not be applied consistently across states. In this regard, The National Health Resource Centre (NHSRC) was entrusted with the task of proposing a standard criterion for defining and determining ‘difficulty’ which could be consistently and objectively applied across all states and to recommend a policy for incentivisation.

The paper draws on the study undertaken by the NHSRC that documented the process of evolving the criteria, validation, the process of negotiation with the states and outcomes in terms of standards for defining the criterion of difficult health facilities.

## Methods

Based on pilot studies, a preliminary set of criteria was developed to define and determine the ‘difficulty’ in four dimensions i.e. physical accessibility, environment (social and physical), housing availability and experience of vacancy. These criteria were applied to assess public health facilities above the sub centre and below the district hospital in 26 states.

Each facility was scored on physical accessibility (A score), environment (E score), housing and family amenities (H score) and vacancy situation (V score) with a numerical score range of 1-5. The score five being the worst or most difficult. The final category for each facility was expressed as a composite score of AEH and V: most difficult being A5, E5, H3 and the least being A1, E0, H0. Then in a process of negotiation with different states, the threshold for difficulty measures was set and additional criteria were added or removed to make it compatible with subjective factors.

## Results and discussion

The study resulted in an extensive database of 26,876 health facilities across 620 districts. This data base had every facility scored for difficulty level by physical distance from an urban area, environmental and social factors, housing and family amenities and experience with vacancy. Initial categorisation of difficulty levels was tweaked with additional or modified criteria to bring it closer to states’ perceptions without losing the objectivity and measurability of the process. The subjective perception of ‘difficult’ matched objective measures of difficulty in over 90% of facilities. However in ten percent, there was no easy resolution. Perceptions of difficulty varied widely across states.

The study categorised the public health facilities into ‘not difficult’ and ‘difficult’ and the latter into three levels of difficulty. The strength of the study is its extensive data base with measures of difficulty for every health facility above a health sub-centre. This allowed the perception of states to be compared with measurable indicators.

Application of a single criterion applied across all states found no health facility as ‘difficult’ in states like Punjab or Tamil Nadu while over one third were considered ‘difficult’ in states like Chhattisgarh or Uttarakhand. While the central government had to prioritise the hilly states, even the better off states needed incentives for what was considered as relatively ‘difficult’ in their contexts. The results of the study recommend that it is important to develop criteria that take into considerations of both standardisation and flexibility, though the experiences of negotiation with states showed varied responses to such a recommendation.

## Competing interests

None declared.

## Funding statement

None declared

